# Genome-Wide Association Studies for *Striga asiatica* Resistance in Tropical Maize

**DOI:** 10.1155/2021/9979146

**Published:** 2021-06-20

**Authors:** Arthur Pfunye, Rwafa Rwafa, Stanford Mabasa, Edmore Gasura

**Affiliations:** Department of Plant Production Sciences and Technologies, University of Zimbabwe, P.O. Box MP 167, Mt Pleasant, Harare, Zimbabwe

## Abstract

*Striga asiatica* L. is a parasitic weed in cereal crops including maize leading to tremendous yield losses up to 100% under severe infestation. The available *S. asiatica* control methods include cultural control options such as uprooting and burning the *Striga* plants before they flower, field sanitation, crop rotation, intercropping, organic matter usage, improved fallows, and application of herbicides. Resource limitation among smallholder farmers renders almost all of the control methods impossible. Development and use of *Striga* resistant genotypes are seen as the most feasible management option. Marker identification formulates tools that are faster, cheaper, and easier to utilise in breeding for *S. asiatica* resistance which has low heritability. The objective of this study was to identify single nucleotide polymorphism (SNP) markers for *Striga* resistance using the genome-wide association study (GWAS). Genotyping by sequencing was done on tropical maize inbred lines followed by their evaluation for *Striga* resistance. Analysis of variance showed significant (*p* < 0.05) variation among evaluated genotypes for *Striga* resistance traits such as germination distance, germination percentage, haustoria root attachments, total *Striga* plants emerged, total biomass, and growth rate. There were also significant differences (*p* < 0.05) for cobs, leaves, stems, and roots weight. The broad sense heritability was fairly high (up to 61%) for most traits. The means for derived traits on stress tolerance indices were subjected to a *t*-test, and significant differences (*p* < 0.05) were found for leaves, stem, roots, shoots, and total biomass. The Manhattan plots from GWAS showed the presence of three SNP markers on chromosome numbers 5, 6, and 7 for total *Striga* plants emerged. The identified markers for resistance to *S. asiatica* should be validated and utilised to breed for *Striga* resistance in tropical maize.

## 1. Introduction

Information about the genetic variation present within and between various populations and their structure can play a valuable role in the efficient utilisation of plants [1]. During the last three decades, the world has witnessed a rapid increase in the knowledge about genome sequences [2] and the physiological and molecular roles of various plant genes, which has revolutionized the applications of molecular genetics in plant breeding programs. Genetic markers are important developments in the field of plant breeding [3]. Genetic markers are closely related with the target gene, and they act as signs or flags of the gene they are associated with [4]. Genetic markers are broadly grouped into two categories, classical and molecular. Classical markers include morphological, cytoplasmic, and biochemical markers [5]. Classical markers are less preferred by breeders because they are less polymorphic and are highly influenced by the environment as well as influenced by the plant growth stage [4]. Molecular markers such as single-nucleotide polymorphism (SNP) markers are the most ideal to use in marker-facilitated breeding because they are codominant, uniformly distributed throughout the genome, highly reproducible, and highly polymorphic [6]. The genome-wide association study (GWAS) is a modern technique used to detect connotation between genetic variants and traits in samples from populations mainly based on SNP markers [7]. This recently discovered technique has led to advancement in the arena of genotyping and sequencing; therefore, it is a powerful technique to study the genetics of natural variation and traits of interest [7]. The trait of attentiveness in this study is *Striga asiatica* resistance in tropical maize.

*Striga* spp. is a parasitic weed in cereal crops including maize leading to tremendous yield losses especially in Africa. In east Africa, there is *Striga hermonthica* while *Striga asiatica* is found in southern Africa. *Striga* resistance is defined as the plant's ability to produce satisfactory grain yield under severe *Striga* infestation, while at the same time carrying fewer flowering *Striga* above the ground [8]. Generally, crops may exhibit the ability to resist *Striga* at any of the growing stages from germination, attachment, subterranean growth, emergence, flowering, and seed production [9]. There are many mechanisms by which crops develop resistance to *Striga* including production of low germination stimulators, growth retardants, and tolerance [10]. Low germination stimulators are based on the low efficiency of the maize in stimulating *Striga* to germinate and a lessened extensive root system of the maize genotype. Growth retardants do not stop *Striga* from germinating and attaching to the roots but rather retard its overall growth, delay emergency, and reduce its vigor. Tolerance is the ability of the host plant to produce high grain yield yet supporting high number of total *Striga* plants emerged [11]. Marker-assisted breeding can give rise to better solution to current problems caused by *S. asiatica* in maize production in sub-Saharan Africa.

The major problem for maize production in Africa is very low yields that are stagnated at around 1.2 tons/hectare/year [12]. Poor agronomic practises, drought stress, pests, and diseases are among other reasons that have been causing low maize yields. However, the ongoing encounter between maize and *S. asiatica* has the furthermost economic bearing [13]. *Striga* is a parasitic weed that attacks maize, resulting in retarded plant growth as well as stunted and withered crops, and reduced grain yield [14]. In severe cases, this leads to 100% yield losses and desertion of fields. The availability of dormant seeds in the seed bank makes *Striga* persistent season after season [15]. The available *Striga* control methods include cultural control methods such as uprooting and burning the problematic weed before flowering, field sanitation, crop rotation, intercropping, organic matter usage, improved fallows, and application of herbicides [15]. Resource limitation among smallholder farmers renders almost all of the control methods impossible. Development and use of *Striga* resistant genotypes are seen as the most feasible management option for *Striga*-related problems [10]. However, little on breeding for *Striga* resistance has been done [11]. A number of efforts were made in east Africa where *Striga hermonthica* is a problem while limited efforts exist in southern Africa where *Striga asiatica* is prevalent [10].

The development of resistance has proven to be strenuous due to its low heritability [16, 17]. Marker identification will formulate tools that are faster, cheaper, and easier to utilise in effectively tackling the problem of breeding for *Striga* resistance [13, 18, 19]. There is a research gap on the use of SNP markers to improve maize genotypes to resist *Striga* infestation [20]. Breeding for *Striga* resistance is the most efficient, cost effective, and environmentally friendly approach to reduce yield loss [10]. There is need to identify SNP markers in maize that are associated with resistance to *Striga* using genome-wide association study (GWAS) because SNP markers are abundantly available in the maize genome, codominant, evenly distributed throughout the genome, and highly reproducible and polymorphic. However, to date, only reports exist for *Striga hermonthica* found in east Africa and absent for *Striga asiatica* found in southern Africa [13, 18, 21, 22]. Identification of SNP markers will solidify convectional breeding methods and bring rejuvenation to maize production in resource poor African farmers by reducing yield losses due to *Striga* infestation [13]. There is need to develop a breeding tool that can be used to breed new varieties that are resistant to *Striga asiatica* based on SNP markers. The aim of this study was to identify SNP markers for *Striga* resistance using the genome-wide association study (GWAS).

## 2. Materials and Methods

### 2.1. Planting Materials

A total of 222 maize inbred lines comprising of 192 inbred lines from International Maize and Wheat Improvement Center (CIMMYT), Harare, Zimbabwe, and 30 inbred lines from International Institute of Tropical Agriculture (IITA), Ibadan, Nigeria, were used in the study (Table 1). The 30 maize inbred lines were bred for resistance to *S. hermonthica* using genes from *Zea diploperennis*, a wild relative of maize but were not yet tested for resistance to *Striga asiatica* in southern Africa.

### 2.2. Genomic DNA Isolation and Genotyping

Seeds were shipped to the Biosciences for East and Central Africa (BeCA) at the International Livestock Research Institute (ILRI) (BeCA-Hub-ILRI) Nairobi, Kenya. The seeds of the 222 inbred lines were germinated, and the DNA was extracted from fresh tissues that were one week old using the modified CTAB method. The DNA was checked for quality using the agarose gel and quantity using a spectrophotometer. Genotyping was done using the genotyping by sequencing (GBS) platform according to the protocol of the Integrated Genotyping Support Services (IGSS) [23] at BeCA-Hub-ILRI, Nairobi, Kenya.

### 2.3. Laboratory Screening Experiment

The agar gel assay was done for *Striga* germination percentage (GP) and *Striga* germination distance (GD). The CIMMYT germplasm was not screened for resistance given that the technique is very difficult to implement in the laboratory and the greenhouse for a large number of genotypes. Furthermore, preliminary evaluations had shown less variability for *Striga* resistance parameters in the CIMMYT germplasm. This may suggest that some of the germplasm sources were from regions without this parasite and lack of coevolution of the host, and the parasite did not allow development of resistance.

*Striga asiatica* seeds collected from different farmer's fields in Rushinga communal area at the end of 2015/2016 rainfall season were preconditioned by placing them on a glass fibre paper and wetted with distilled water in a 9 cm diameter Petri dish that were sealed using cello tape and wrapped in a black polythene paper to exclude light and were kept in the glass house for 28 days. The *S. asiatica* seeds were surface sterilised by immersing them in 1% sodium hypochlorite for 30 minutes before setting up the assay. The assay setup was done following the standard procedures [11], but with slight modifications as follows. A total of 150 *μ*l of preconditioned *S. asiatica* seeds were pipetted into eight (9 cm diameter) Petri dishes using a 50 *μ*l micropipette. A total of 90 ml of autoclaved water agar was poured into the Petri dishes containing *Striga* seeds. The agar was poured just before it became cool enough to solidify.

A seed to represent each maize genotype pregerminated by soaking in water for 48 hours was submerged in the solidifying agar at the edge of the dish with radicle pointing across the Petri dish. Each genotype had three replicates. The Petri dishes were then incubated for 48 hours, at 27°C. After 48 hours, the Petri dishes were examined using a dissecting microscope at ×100 magnification to observe germination of the *Striga* seeds. *Striga* seed observed to be germinating at the closest distance from the maize was recorded and used as the index for *Striga* germination. A Petri dish lid graduated with 32 (1 mm × 1 mm) squares was used to count the number of germinating *Striga* seeds out of the seeds under focus in one square and expressed as a percentage.

Furthest germination distance was measured and recorded. The number of germinated seeds as a percentage of the total number of seeds visible in a given quadrant under a light microscope was also recoded per each genotype.

### 2.4. Greenhouse Experiment

One hundred and eighty-six asbestos pots measuring 15 cm diameter and 20 cm height were filled with fine sand soil up to three-quarter full. In 50% of the pots, the top seven centimetres of dry soil was thoroughly mixed with 0.05 g of *S. asiatica* seeds (approximately 12,250 seeds). Mixing of *S. asiatica* seeds with soil was done by shaking the soil with *S. asiatica* seeds in a polythene bag. Planting of maize was done on the 16^th^ of December 2018. One seed was planted in moist soil seven centimetres deep per pot for all the maize genotypes both in the infested and uninfested pots. The top seven centimetres was mixed with 2 g/pot of compound D. The maize emerged on the 23^th^ of December 2018.

The pots were watered daily using a watering can fitted with a fine rose to keep the pots moist. Other weeds were removed from the pots by hand pulling to allow interactions between maize and *Striga* only. Ammonium nitrate (34.5% N) was applied in the splits of 0.8 g at four weeks after planting (WAP), 0.8 g at six WAP, and 0.4 g at 10 WAP to achieve an application rate of 30 kg N/ha.

Data was collected on a number of traits. The number of total emerged *Striga asiatica* plants per pots was recorded at weekly interval from nine weeks of planting to harvesting. The total number of *S. asiatica* haustoria root attachments was counted at maturity. Maize roots were washed by gently immersing the root ball in a large bucket of water, and decaying old attachments were counted and weighed as well. Maize plant height was measured from the base of the stem up to the ligule of the last fully expanded leaf at weekly intervals from three WAP to 10 WAP. Above-ground biomass included stem, leaf, and cob biomass. After cutting the maize plant at the base of the stem using secateurs, the leaves and the cobs were removed from the stem and dried in khaki envelops in an oven at 80°C for 72 hours and then weighed using a digital balance. Thoroughly washed maize roots were put in a khaki envelop paper and oven dried at 80°C for 72 hours and then weighed using a digital balance. The root-to-shoot ratio was calculated as weight of oven-dried roots divided by weight of dried total above-ground biomass. The stress tolerance index was calculated as the difference between uninfested and infested observations divided by the observation under uninfested conditions [24].

### 2.5. Data Analysis

A total number of 45,000 SNP markers were sampled in this study. The RStudio software was then used for cluster analysis to depict possible groups using Gower's distance and neighbor joining algorithm [25]. Not all of the 222 materials were evaluated for *Striga* resistance. Analysis of variance (ANOVA) for different maize parameters recorded was done using GenStat software 18^th^ edition. Mean separation was done using the 5% Fischer's protected least significant difference (LSD). Although there were significant differences among materials used, there were no significant variation among the CIMMYT materials sampled and tested, but variation existed among the IITA materials (Table 2). In this regard, only the phenotypic traits of the IITA materials are presented. Genome-wide association studies were done between phenotypic and genotypic data using the Genome Association and Prediction Integrated Tool (GAPIT) package in RStudio [25].

## 3. Results

There were highly significant differences (*p* < 0.001) among genotypes for germination percentage and germination distance (Table 2), with some genotypes having fairly low mean values (Table 3). There were significant differences (*p* < 0.05) among inbred lines for total *Striga* plants emerged and haustoria root attachment (Table 4).

An unbalanced combined ANOVA including inbred line, number of replicates, and environment factor as either infested or uninfested showed highly significant (*p* < 0.05) differences for cob, leaf, roots, shoots, stem, total biomass, and growth rate (Table 5). The growth rate and total biomass were significantly (*p* < 0.05) affected by conditions (infested against uninfested) and inbred line. Lower heritability values were observed for stem, and fairly high heritability values were observed for cob, leaf, root, shoots, total biomass, and growth rate.

Data was sorted for each trait from minimum value to maximum value and was divided at the centre into two groups, A and B. A *t*-test analysis for the unpaired groups was done, and significant differences (*p* < 0.05) were found for leaves, roots, shoots, and total biomass (Table 6).

Interestingly significant (*p* < 0.05) positive correlations were found for some trait such as leaf biomass tolerance index and stem biomass tolerance index, and shoot biomass tolerance index and root biomass tolerance index (Table 7). Correlation was 100% for height and growth rate because growth rate was calculated using plant height.

## 4. Discussion

**Figure 1:**
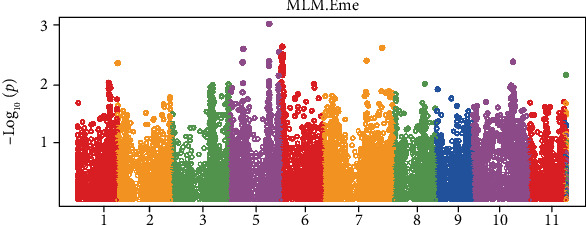
The Manhattan plot for total *Striga* plants emerged shows that there are three SNP markers at chromosomes 5, 6, and 7 considering a LOD score of 2.5.

The existence of genetic variation in *Striga* resistance parameters among maize materials indicated the potential to use this variation in the identification of markers associated with *Striga* resistance. Indeed, markers at chromosomes 5, 6, and 7 for total *Striga* plants emerged were identified, and these could be validated and used in future breeding programs (Figure 1). The three SNP markers found for total *Striga* plants emerged suggest the feasibility of making selections effectively at genomic level. The ability of *Striga* to emerge shows the compatibility of the host and parasite. In resistant cases of incompatibility, the number of *Striga* plants that emerge would be greatly reduced or can be absent at all [10, 13]. Information on quantitative traits loci for *Striga* resistance is very scarce [18, 21, 26]. Adewale et al. [21] identified a set of 24 SNP markers for various traits including *Striga* damage rating in maize inbred lines from east Africa while working on *Striga hermonthica*. Markers for *Striga* counts and *Striga* damage were found on chromosome 1, 3, 7, 8, 9, and 10. The number of emerged *Striga* plants should be related to the extent of the associated damage.

Interestingly, in this study, markers for total *Striga* plants emerged were found on chromosome 7 just like markers for *Striga* damage rating which have been also found by Adewale et al. [21] to be located on several chromosomes including number 7. Working on *Striga hermonthica*, Gowda et al. [22] also found SNP markers for emerged *Striga* plants and *Striga* counts at chromosomes 5 and 7, respectively. These findings are in line with the observations from this study that also found the markers total *Striga* plants emerged at chromosomes 5, 6, and 7 for *Striga asiatica*, a strain that is found in southern Africa.

At the moment, there are no maize varieties specifically bred for *Striga asiatica* resistance in southern Africa [11]. However, various resistance mechanisms to *Striga asiatica* have been reported in maize inbred lines of tropical origin [10]. These resistance mechanisms could be exploited in breeding better varieties; however, the heritability for *Striga* resistance is low [16, 17]. When heritability is low, the use of molecular markers is usually effective [22]. The SNP markers identified in this study for the total number of *Striga* plants emerged could thus go a long way if they are validated and utilised in breeding programs.

Agriculture has faced tremendous challenges in crop production in the past decades due to many abiotic and biotic factors such as parasitic weeds. In the future, it is predicted that there will be a further reduction in crop production due to predicted changes in temperature and rainfall patterns. Breeding for resistance to environmental, weeds, insects, and disease stress in plants is the most appropriate strategy in plants especially targeting yield increment among resource poor farmers. *Striga asiatica* plague is one of the major yield reduction factors in Africa among resource poor farmers [13]. Efforts must be made in the region to develop maize varieties that are resistant to such parasitic weeds.

The development of new varieties using the convectional breeding approach is usually labour-intensive and slow [22]. New breeding methodologies have been proposed with the advent of molecular markers. However, for those molecular markers to be developed, phenotypic screening is also required for different resistance mechanisms [10]. Modern phenotypic screening tools that incorporate remote sensing could help reduce the human error in experiments. However, these tools are yet to be developed for *Striga* resistance breeding thus necessitating again the need for molecular screening.

Phenotypic screening is subject to many challenges. The plant reacts by exhibiting phenotypic plasticity when the genotype is grown under various environmental conditions, and this plasticity is particularly big under extreme stress conditions such as *Striga* infestation. Phenotyping in general is labour-intensive, time-consuming, and costly and requires destruction of plants at fixed times or at particular phenological stages. A further source of variation can be introduced by human error, and for *Striga* breeding, incorporation of modern phenomics is encouraged.

Most traits had positive correlations. Correlations indicated that some traits can be used for phenotypic selection for grain yield under *Striga* stress. There are many reports in which indirect selection was done via other traits in breeding [27, 28]. The use of molecular markers would not replace phenotypic screening in breeding. There is therefore a need to integrate the various screening tools ranging from phenomics to genomics.

## 5. Conclusion

Three SNP markers have been identified on chromosomes 5, 6, and 7 for total *Striga* plants emerged. These makers can be validated and used in marker-assisted breeding or in genomic prediction for *Striga asiatica* resistance.

## Figures and Tables

**Table 1:** Maize inbred lines from IITA used in the study.

Entry number	Genotype names
2	TZISTR1116
3	TZISTR1172
4	TZSTR170
5	IITATZISTR1132
6	TZISTR1119
7	TZISTR1175
8	TZSTR179
9	IITATZISTR1137
10	TZISTR1133
11	TZISTR1178
12	TZSTR182
13	IITATZISTR1138
14	TZISTR1134
15	5057
16	TZSTR184
17	IITATZISTR1156
18	TZISTR1154
19	9540
21	TZSTR186
22	IITATZISTR1157
23	TZISTR1160
26	IITATZISTR1158
27	TZISTR1162
29	TZSTR189
30	IITATZISTR1159
31	TZISTR1166
33	IITATZSTR194

**Table 2:** Mean squares and genetic parameters for germination distance and germination percentage.

Source of variation	DF	Germination distance	Germination percentage
Replications	3	32.31	628.9
Inbred lines	29	258.43^∗∗∗^	679.1^∗∗∗^
Residual	90	49.49	268.1
IVC		52.24	102.75
EVC		49.49	268.1
VP		101.73	370.85
BSH		51.35	27.71
GM		17.59	40.08
GCV		41.09	25.29
PCV		57.34	48.05

DF: degrees of freedom, IVC: inbred line variance component, EVC: error variance component, VP: phenotypic variance, BSH: broad sense heritability, GM: grand mean, GCV: genotypic coefficient of variation, PCV: phenotypic coefficient of variation. ^∗∗∗^ means significant at *p* ≤ 0.001.

**Table 3:** Effects of maize genotypes on *Striga asiatica* seed germination distance and germination percentage.

Inbred line	Germination distance	Inbred line	Germination percentage
18	1.67^a^	11	0^a^
27	3^a^	27	0^a^
20	4^a^	32	0.33^a^
32	4.67^a^	20	0.67^a^
31	8.33^a^	18	1.67^a^
34	9.33^a^	23	5.33^a^
1	13^a^	16	5.33^a^
23	16.67^ab^	13	5.67^a^
11	19.67^ab^	1	6.33^a^
16	21.33^ab^	2	6.67^a^
19	31.67^ab^	25	6.67^a^
5	32.33^ab^	28	6.67^a^
22	33.33^ab^	5	7.33^a^
24	35.33^ab^	31	7.67^a^
2	35.67^ab^	21	7.67^a^
10	36.33^ab^	34	8.33^a^
13	37.67^ab^	4	9^a^
28	42^ab^	33	9^a^
7	43.67^ab^	15	11^a^
14	54^ab^	19	11.33^a^
4	55^ab^	22	11.67^a^
9	55.33^ab^	14	12^a^
3	60^ab^	7	12.33^a^
15	60.67^ab^	24	14.33^a^
33	65.67^ab^	29	18^a^
29	75.67^ab^	10	21.33^a^
21	90.67^ab^	9	23.33^a^
25	123.67^ab^	26	23.67^a^
26	150^ab^	3	24^a^
17	169.33^b^	17	26.33^a^
Mean	46.32	Mean	10.12
5% LSD	75.78	5% LSD	15.18

LSD: least significant difference.

**Table 4:** Mean squares for total *Striga* plants emerged and haustoria root attachment.

Source of variation	DF	Total *Striga* plants emerged	Haustoria root attachment
Replications	2	145.88	5332
Inbred lines	29	169.14^∗^	5195^∗∗^
Residual	58	86.29	2150
IVC		20.71	761.25
EVC		86.29	2150
VP		107	2911.25
BSH		19.36	26.15
GM		10.12	46.32
GCV		44.96	59.56
PCV		102.2	116.48

DF: degrees of freedom, IVC: inbred line variance component, EVC: error variance component, VP: phenotypic variance, BSH: broad sense heritability, GM: grand mean, GCV: genotypic coefficient of variation, PCV: phenotypic coefficient of variation. ^∗^ and ^∗∗^ means significant at *p* ≤ 0.05 and *p* ≤ 0.01, respectively.

**Table 5:** Mean squares and heritability for morphological traits and growth rate under stress and non-stress conditions.

SOV	DF	Cob	Leaf	Roots	Shoots	Stem	Total biomass	Growth rate
Rep	2	277.9	27.82	741.9	802.2	45.96	867	12.09
Rep. block	27	178.1	22.54	579.6^∗∗^	524.7	73.87∗	3116	26.65
*Striga*	1	2809.1^∗∗∗^	70.25	22,381.5^∗∗∗^	8757.5^∗∗∗^	1036.77^∗∗∗^	1411	414.53^∗∗∗^
Inbred	29	375.6^∗∗^	31.29^∗^	543^∗∗^	862.8^∗^	74.71^∗^	4975^∗∗^	43.21^∗∗∗^
Inbred x *Striga*	29	174	18.65	427.2^∗^	460.4	75.29^∗^	2210	14.47
Residual	87	186.8	18.83	242.3	466.7	45.81	2181	16.71
Total	175	229.7	21.77	501.1	588.7	65.04	2760	24.48
V error		186.8	18.83	242.3	466.7	45.81	2181	16.71
Var GE		0	0	61.63	0	9.83	9.67	0
Var G		31.47	2.08	50.12	66.02	4.82	465.67	4.42
VP		218.27	20.91	354.05	532.72	60.45	2656.33	21.13
Mean		23.43	11.03	19.2	49.9	14.49	119.1	35.3
GCV		23.94	13.06	36.87	16.28	15.15	18.12	5.95
PCV		63.06	41.45	98	46.25	53.66	43.27	13.02
Heritability		0.50	0.40	0.41	0.46	0.28	0.56	0.61

SOV: source of variation, DF: degrees of freedom, V error: variance for error, VGE: variance for genotype × environment interaction, VG: variance for genotype, VP: phenotypic variance, GCV: genetic coefficient of variation, PCV: phenotypic coefficient of variation. ^∗^, ^∗∗^, and ^∗∗∗^ mean significant at 0.05, 0.01, and 0.001probability levels, respectively.

**Table 6:** A *t*-test analysis for stress tolerance indices for the derived traits.

	GRTI	CobTI	LeafTI	RootTI	ShootTI	StemTI	TbioTI
Group A mean	18.15	15.73	19.5	9.07	18.91	12.27	22
Group A size	13	11	8	14	11	11	7
Variance for group A	104.8	80	6	29.9	93.6	17.8	4.6
Standard error of group A	2.83	2.69	0.86	1.46	2.91	1.27	0.81
Group B mean	17.82	18.36	28.9	25.75	16.69	26.27	30
Group B size	17	11	10	16	13	15	8
Variance for group B	99.9	100.2	12.1	26	86.2	23.3	8.5
Standard error of group B	2.42	3.01	1.1	1.27	2.57	1.24	1
Mean differences	0.33	-2.63	-9.4	-16679	2.21	-13.99	-8
Standard error of the difference	3.72	4.04	1.45	1.93	3.87	1.82	1.34
*t*-value	0.09	-0.65	-6.45	-8.64	0.57	-7.68	-5.94
Degrees of freedom	28	20	16	28	22	24	13
*t*-probability	0.93	0.522	<0.001	<0.001	0.573	<0.001	<0.001

GRTI: growth rate tolerance index, CobTI: cob biomass tolerance index, LeafTI: leaf biomass tolerance index, ShootTI: shoot biomass tolerance index, StemTI: stem biomass tolerance index, TbioTI: total biomass tolerance index.

**Table 7:** Correlations among *Striga* resistance parameters.

GRTI	1							
HTI	1.00^∗∗∗^	1						
LTI	0.23	0.18	1					
RSRTI	-0.41	-0.12	-0.36	1				
RTI	-0.18	-0.06	0.56^∗∗^	0.2	1			
ShTI	0.41^∗^	0.40^∗^	*0.75* ^∗∗∗^	-0.43	0.27	1		
StTI	0.22	0.2	*0.61*∗∗∗	-0.3	0.34	*0.83* ^∗∗∗^	*1*	
TBTI	0.26	0.32	*0.81* ^∗∗∗^	-0.27	*0.60* ^∗∗∗^	*0.91* ^∗∗∗^	*0.79* ^∗∗∗^	*1*
	GRTI	HTI	LTI	RSRTI	RTI	ShTI	StTI	TBTI

GRTI: growth rate tolerance index, HTI: height tolerance index, LTI: leaf biomass tolerance index, RSRTI: root-to-shoot biomass ratio tolerance index, RTI: root biomass tolerance index, ShTI: shoot biomass tolerance index, StTI: stem biomass tolerance index, TBTI: total biomass tolerance index.

## Data Availability

The phenotypic and genotypic data used to support the findings of this study are included within the supplementary information files (available here).

## Supplementary Materials

Supplementary Materials Table S1: the phenotypic data file contains the raw data collected on various *Striga* resistance traits on 30 maize inbred lines from IITA. Table S2: the genotypic data file contains the HapMap genotypic information for the 30 maize inbred lines from IITA. The two data files can be used to run genome-wide association studies (GWAS), and the GAPIT package in the R software was used by the authors.
