# Supplementary material for: Genome-Wide Association Studies for Striga asiatica Resistance in Tropical Maize
Source: Int J Genomics. 2021 Jun 20;2021:9979146. doi: 10.1155/2021/9979146 (PMC8238628; doi:10.1155/2021/9979146)
Supplement: Supplementary Materials — Table S1: the phenotypic data file contains the raw data collected on various Striga resistance traits on 30 maize inbred lines from IITA. Table S2: the genotypic data file contains the HapMap genotypic information for the 30 maize inbred lines from IITA. The two data files can be used to run genome-wide association studies (GWAS), and the GAPIT package in the R software was used by the authors. [file 9979146.f1.zip › 9979146.f1/Phenotypic data.docx]

| Inbred line name | Inbred line code | Emergence | Germination percentage | Haustorium root attachment | Germination distance | Height | Growth rate | Cob mass | Shoot mass | stem mass |
| --- | --- | --- | --- | --- | --- | --- | --- | --- | --- | --- |
| TZISTR1116 | 1 | 7.67 | 29.97 | 90.67 | 14.5 | 15 | 0.392445 | 0.461538 | 0.536451 | 0.603976 |
| TZISTR1172 | 2 | 18 | 29.67 | 75.67 | 7.5 | 22.8 | 0.36573 | 0.0949 | 0.06945 | 0.284117 |
| TZSTR170 | 3 | 6.33 | 35.8 | 13 | 15 | 15.3 | 0.171227 | 0.74847 | 0.54382 | 0.41267 |
| IITATZISTR1132 | 4 | 5.67 | 49.05 | 37.67 | 14.5 | 28.9 | 0.37329 | 0.685551 | 0.470552 | 0.30398 |
| TZISTR1119 | 5 | 11.67 | 25.15 | 33.33 | 12.5 | 16.3 | 0.150217 | 0.073658 | 0.1508 | 0.372446 |
| TZISTR1175 | 7 | 7.67 | 55.97 | 8.33 | 31 | 30.5 | 0.407273 | 0.06228 | 0.01245 | 0.100617 |
| TZSTR179 | 11 | 6.67 | 12.8 | 35.67 | 1.5 | 25.9 | 0.421009 | 0.297475 | 0.090696 | 0.011918 |
| IITATZISTR1137 | 13 | 12 | 18 | 54 | 10 | 16.5 | 0.102352 | 0.347142 | 0.351801 | 0.462302 |
| TZISTR1133 | 14 | 5.33 | 45.22 | 16.67 | 19 | 51.3 | 0.465653 | 0.510985 | 0.376765 | 0.249289 |
| TZISTR1178 | 15 | 0.33 | 40.55 | 4.67 | 5 | 18.7 | 0.23136 | 0.043889 | 0.063757 | 0.138286 |
| TZSTR182 | 17 | 24 | 54.62 | 60 | 30.25 | 50.2 | 0.628625 | 0.63399 | 0.388685 | 0.304032 |
| IITATZISTR1138 | 18 | 11 | 57.15 | 60.67 | 22 | 51.5 | 0.646196 | 0.256537 | 0.4227 | 0.667932 |
| TZISTR1134 | 19 | 14.33 | 41.85 | 35.33 | 23.5 | 11.8 | 0.188736 | 0.23934 | 0.09615 | 0.085183 |
| 5057 | 20 | 9 | 23.9 | 65.67 | 8 | 18.5 | 0.312652 | 0.948705 | 0.64757 | 0.529769 |
| TZSTR184 | 21 | 9 | 62.57 | 55 | 29.25 | 16.4 | 0.252435 | 0.29123 | 0.17052 | 0.021087 |
| IITATZISTR1156 | 22 | 26.33 | 46 | 169.33 | 24.75 | 37 | 0.373556 | 0.51086 | 0.351932 | 0.367448 |
| TZISTR1154 | 23 | 6.67 | 27.72 | 123.67 | 26.75 | 31.6 | 0.390585 | 0.40515 | 0.337655 | 0.446071 |
| 9540 | 24 | 8.33 | 51.22 | 9.33 | 29.25 | 67 | 0.544237 | 0.877819 | 0.818444 | 0.774648 |
| TZSTR186 | 25 | 7.33 | 47.9 | 32.33 | 20.25 | 22.4 | 0.327133 | 0.240362 | 0.260334 | 0.39626 |
| IITATZISTR1157 | 26 | 1.67 | 48.37 | 1.67 | 24.75 | 41.6 | 0.334862 | 0.32943 | 0.349737 | 0.43684 |
| TZISTR1160 | 27 | 23.67 | 40.22 | 150 | 18.25 | 48.9 | 0.359587 | 0.372148 | 0.00866 | 0.40541 |
| IITATZISTR1158 | 28 | 11.33 | 54.47 | 31.67 | 17.75 | 13.5 | 0.092249 | 0.20626 | 0.218599 | 0.221245 |
| TZISTR1162 | 29 | 0 | 21.5 | 3 | 8.5 | 8.5 | 0.038869 | 0.00261 | 0.043707 | 0.080724 |
| TZSTR189 | 31 | 12.33 | 32.12 | 43.67 | 9.75 | 10.2 | 0.08833 | 0.563722 | 0.477956 | 0.494327 |
| IITATZISTR1159 | 32 | 0.67 | 48.7 | 4 | 20.75 | 3.2 | 0.02046 | 0.58025 | 0.151857 | 0.596373 |
| TZISTR1166 | 33 | 6.67 | 22.87 | 42 | 6.25 | 7.6 | 0.028595 | 0.25712 | 0.58746 | 1.27124 |
| IITATZSTR194 | 34 | 0 | 52.92 | 19.67 | 17.25 | 26.4 | 0.298343 | 0.041644 | 0.076881 | 0.51661 |
